# A MicroRNA Perspective on Cardiovascular Development and Diseases: An Update

**DOI:** 10.3390/ijms19072075

**Published:** 2018-07-17

**Authors:** Jose Francisco Islas, Jorge Eugenio Moreno-Cuevas

**Affiliations:** Tecnologico de Monterrey, Grupo de Investigación con Enfoque Estratégico en Bioingeniería y Medicina Regenerativa, Escuela de Medicina y Ciencias de la Salud, Ave. Morones Prieto 3000, Monterrey, NL 64710, Mexico; chepo@mail.com

**Keywords:** microRNA, cardiovascular diseases, myocardial infarction, atherosclerosis, heart failure

## Abstract

In this review, we summarize the latest research pertaining to MicroRNAs (miRs) related to cardiovascular diseases. In today’s molecular age, the key clinical aspects of diagnosing and treating these type of diseases are crucial, and miRs play an important role. Therefore, we have made a thorough analysis discussing the most important candidate protagonists of many pathways relating to such conditions as atherosclerosis, heart failure, myocardial infarction, and congenital heart disorders. We approach miRs initially from the fundamental molecular aspects and look at their role in developmental pathways, as well as regulatory mechanisms dysregulated under specific cardiovascular conditions. By doing so, we can better understand their functional roles. Next, we look at therapeutic aspects, including delivery and inhibition techniques. We conclude that a personal approach for treatment is paramount, and so understanding miRs is strategic for cardiovascular health.

## 1. MicroRNA’s

Initial studies by Ruvkon et al. and Ambros et al. in the 1990s demonstrated that a group of small non-coding RNAs influenced the development of *Caenorhabditis elegans* by regulating translation thorough a process of base-pairing (inhibiting translation) to the 3′UTR and, in a few cases, the 5′UTR of mRNA [1,2]. These small RNAs, termed microRNAs, were over time shown to be conserved in many cellular eukaryotic species including human cells [2,3,4,5].

MicroRNAs (miRs), commonly designated *miR* for the precursor product and miR for the mature product, are a set of 18–24 nucleotides which, when processed (a process of complementary base pairing to mRNA), can silence or downregulate expression of their targets. Their function is to either destabilize and/or cleave the target mRNA, or reduce the efficiency with which the mRNA can be processed [6,7]. Unlike other small RNAs, miRs have a predictable hairpin loop structure from their precursor transcript [8]. This small region, required for miRNA regulation, is only about eight nucleotides long. Such a small region, potentially homologous in many places, suggests that one miR regulates as much as 60% of available mRNAs, although this has been found not to be the case [9]. MiRNA translation regulation is a tightly regulated mechanism that is controlled in a spatial-temporal manner, meaning that a handful of miRs can be secreted in specific organs and are taken up far from their origin [5,6,10].

### miRNA Biogenesis

**Figure 1:**
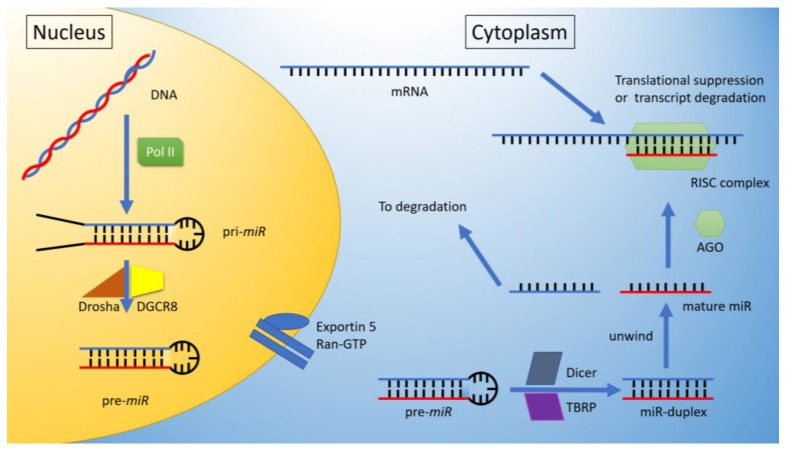
mRNA biogenesis. Transcription of a pri-*miR* by pol II, followed by cleavage (Drosha/DGCR8 complex) to a pre-*miR*. The pre-*miR* is later exported to the cytoplasm via exportin-5/Ran-GTP, where it can be further cleaved by a Dicer/TRBP complex and unwind into its mature form. This is further packed by AGO into the RNA-induced Silencing Complex (RiSC) with mRNA. The consequence of such loading is either a transcriptional suppression or transcript degradation.

Briefly, miRs are initially transcribed from the genomic region by pol II (up to 3 kb) as a combined primary-precursor *miR* (pri-*miR*). Further processing by Drosha/DGCR8 permits cleavage into a ~70–100 bp stem-loop hairpin, or precursor-*miR* (pre-*miR*). The pre-*miR* is exported to the Cytoplasm; a process of the pre-*miR* binding to Exportin-5, followed by a complexing with Ran-GTPase, promoting exporting through the nuclear pore [1]. Once exported, further enzymatic cleavage by Dicer/TRBP complex produces miR duplexes (22 bp long). These duplexes serve as the guides that pair with mRNA; this is achieved initially by a dissociation of the duplexes, followed by assimilation of one of the miR strands with Argonaute (AGO), thus forming the RNA-induced Silencing Complex (RiSC), which consequently binds to the complementary sequence of the mRNA. Typically, binding is to the 3′UTR of the mRNA, promoting posttranslational degradation or downregulation of expression [1,11,12,13,14] (see Figure 1).

Given the above, it is no surprise that miRs are such important regulatory molecules. In many instances, they self-regulate by co-expression with target genes. As a result, many research groups have dedicated a considerable amount of time and resources to studying and profiling miRs and their role in development, as well as diseases caused by miR mis-expression, errors in transcription, promoter defects (such as hypo- or hyper-methylation), and others [5,8,15,16,17,18]. Note that the remainder of this review refers to the functional properties of miRs.

## 2. miRNAs in Cardiomyocytes

Fundamentally, the adult heart has little potential to regenerate when suffering injury or disease. Normally, cardiomyocytes are lost, leading to heart failure and, in more complicated cases, death [19,20,21]. Hence, understanding cardiomyocyte development has been key to understanding repair.

Studies of individual miRs using developmental models of the heart have led to the discovery that miR-1/miR-133 are fundamental to both the control of proliferation as well as the regulation of muscle transcriptional networks, e.g., *SRF, MEF2c, MyoD, Hand2*, and *Myocardin*. Interestingly, SRF has been found to be a requirement for miR-1 expression during development. This double mechanism is particularly evident, as SRF/miR-1 also come together to regulate the sodium–calcium exchanger *NCX1* [22]. Additionally, miR-1 overexpression blocks ventricle myocyte expansion [23]. Furthermore, miR-1 has been linked to the NOTCH1 receptor. Its ligand, Dlk-1, is a critical factor in specification through asymmetric division [24]. Mouse studies by Wu et al. found evidence that miR-34a is a repressive regulator to NOTCH1, while upregulating *Jagged1, Hey2,* and *Hes* [25]. A variant of miR-1-1 in mice is miR-1-2, and its deletion has been found to be lethal to approximately 50% of embryos, while around 20% of survivors have major cardiac defects. Moreover, its deletion has also been reported to repress *Kcnd2*, a crucial factor in repolarization of the heart [26,27,28]. In adult rat experiments, miR-1 has been also revealed to target *KCNQ1* and *KCNE1* [18].

Meanwhile, the miR-17-92 cluster has been implicated in cardiac proliferation by negatively regulating the *PTEN* tumor suppressor gene [9,29,30]. Additional reports suggest that miR-17-92 cluster overexpression could cause *Tsc1* repression. Consequently, it causes mTOR-mediated hypertrophy, inferring that the conditional downregulation of Tsc1 acts as a negative regulator of mTOR and a downstream target of PTEN [31]. Furthermore, the miR-15 family seems to balance proliferation by repressing cell cycle regulators, in particular through decreasing *HSP-20*, targeting *Bcl2*, and repressing *TGFβ* activity [30].

Finally, a distinguishing trait of cardiomyocytes is their contraction. This is provided in part by a delicate balance of α/β-*MHC*, chiefly controlled by miR-208. Initially, miR-208a was found to regulate *GATA4* and *CX40*, thereby partially regulating the conduction system. In addition, Callis et al. found that both isoforms of miR-208 (a/b) target *TRAP1* and *myostatin*, which are important negative regulators of muscle growth and hypertrophy [32,33] (a synopsis of knowledge about miR regulation during heart development is shown in Table 1).

### miRNAs in Stem Cells to Cardiomyocyte Differentiation

In order to bypass limitations for cardiomyocyte regeneration, techniques such as direct differentiation of somatic cells, and use of induced pluripotent stem (IPS) cells and embryonic stem (ES) cells, have been the focus for many groups for the past two decades, and still continue to be a large field of study within organ repair [33,41].

Direct differentiation of fibroblasts to cardiomyocytes was initially achieved using the transcription factors Hand2, GATA4, MEF2c, and TBX5 [42], and later enhanced by the addition of MYOCD, SRF, SMARCD3, and MESP1, yet they never addressed the role of miRs [33,39,43]. Nonetheless, by pioneering the use of miRs, Dzau´s group began to show the potential of miR-1, by demonstrating that its overexpression was sufficient to convert cells [40]. 

In ES cells, miR-1/-133 collectively induce mesoderm formation. During this stage both, miR-199 and miR-483 are induced [33]. In addition, Srivastava’s group demonstrated that suppression of both endoderm and neuroectoderm at E4 (EB) results in the repression of Dlk-1, consistent with the expression and possible dependency of *twitst* [34]. Moreover, by expressing miR-1/-133 on SRF null ES cells—known to block muscle differentiation—cardiac differentiation was achieved (no sarcomeres could be observed) [20,34,35].

Expression of miR-1/-499 in ES cell-derived cardiomyocytes predominantly controls the electrical/conduction system. Their expression upregulates *Kir2.1, Kv1.4, HERG,* and *DHPR* while downregulating *HCN4*, resulting in cardiac potassium current channel increase I_to_, I_ks_, and I_kr_, and decreased I_f_ [18,38], a result consistent with the expression of the RYR2 L-type channel [44]. In particular, miR-499 has been implicated in the expression of *MHC6, MHC7, MLC2,* and *TNNT2* [33].

**Figure 2:**
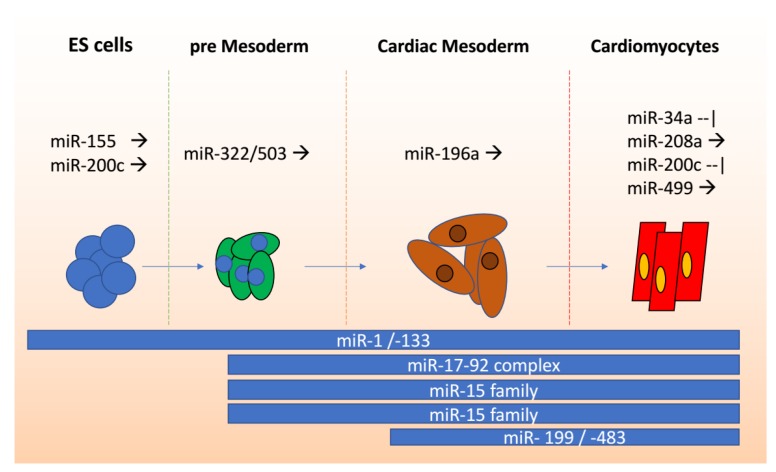
Embryonic Stem cells to cardiomyocytes. Left to right, the tight regulation of miRNAs during ES cell differentiation (blue) to cardiomyocytes (red) including pre- (green) and mesoderm stages (brown). The top shows important miRNA activators (black arrows) and repressors (black stops) at specific stages. The bottom part (in blue lines) shows continuous miRNA activation during prolonged stages.

Poon et al. recently described the importance of miR-200c using ES cells, denoting *GATA4, TBX5*, and *SRF* to be its targets [36]. They noted that knockouts in ES cells altered Ca^+^, Na^+^, and K^+^ ion channels (*CACNA1C, KCNJ2*, and *SCN5A*), increasing contractility [45]. Additionally, conduction seems to be altered if miR-1(-2) is deleted, via dysregulating the expression of *Irx 4, 5*, and *Kcnd2*. Meanwhile, transcription factor MESP1, once described as the key regulator of the heart by Bondue and Blanpain (2010), regulates *Nkx2.5, Tbx5, Hand2, FoxH1, Isl1*, and others, while at the same time inducing mesoderm lineage by blocking *Bry, Fgf6, FoxA2, Sox17*, and *Gsc* [46]. Thereby, *MESP1* sits atop of the hierarchy of cardiomyocyte formation and regulation [47]. At an earlier stage, we find that transactivation is effected by miR-322/503 by targeting the RNA binding factor *Celf1*, which would otherwise induce a neural fate [37]. Regulation via the most important miRs during differentiation can be seen in Figure 2.

Comprehending the role of miRs in all aspects of the cardiovascular system is in itself a formidable task that warrants further in-depth discussion in a variety of areas. The goal of this review is to inform the reader about a handful of clinically relevant diseases in which miRs not only play a crucial role but in which their directive could be key in regulating or attenuating them. We will also briefly allude to other aspects of cardiac miR regulation. Concisely, miR-21’s function during stress is one of the most studied. It is known that miR-21 up-regulation represses *Spry1* [48,49], leading to PI3K/Akt signaling and increased Matrix metallopeptidase 2 (*MMP-2*) expression via *PTEN* repression. As a corollary, TGF-β1 seems to be regulated by Spry1, also as a response to Ang II in atrial fibroblasts [49,50]. Fibrosis, an outcome of these signaling cascades, seems to also be exacerbated by the fact that miR-21, encapsulated in exosomes, is excreted by the proliferating fibroblasts [48,51]. Moreover, the family of miR-29 seems to be the opposite. During and after cardiovascular disease stress, the miR-29 family peaks in its function, promoting expression of collagens, fibrilins, and elastins—all factors involved in fibrotic proliferation. Its knockdown has been independently confirmed to upregulate collagens in the heart [5,9]. Another interesting member is miR-133, which maintains a balance in the expression of connective tissue. Thus, downregulation of this factor stimulates the extracellular matrix [9]. In addition, it seems to be co-expressed with miR-30. Studies have shown that during hypertrophy or heart failure; the expression of both factors is decreased alongside connective tissue growth factor [48,52].

Inflammatory response in the heart is characterized by a myriad of events in which monocytes, macrophages, and leukocytes participate. As a result, there is a production of pro- and anti-inflammatory cytokines such as Tumor necrotic factor α (TNF-α), and Interleukin 1, 6 (IL-1, -6) [53,54]. MiRs are involved in the signaling cascades, of which some of the best studied include miR-155 and miR-233, which inhibits *NF-κβ* via repression of *IKK-β*. Systemic administration has been proven to mitigate dysfunction and improve survival by targeting *JNK*. Nevertheless, miR-155 knockdown triggers apoptosis via induced liposaccharides [45,55,56]. In concert with miR-155, miR-146 downregulates expression of *IRAK1* and *TRAF6*, which are upstream activators of NF-κβ [5]. Additionally, IL-6 and collagen expression appears to be regulated by let-7i-5p. Let-7i-5p activation reduces general inflammation by downregulating *IL-6. IL-6*, along with *NLRP3*, is mediated by miR-233 in monocytes [45]. Further, during myocarditis, miR-155 upregulates macrophages and CD4^+^ T lymphocytes [55]. Another strategic player during inflammation is miR-126, due to its control over endothelial *VCAM-1*, which in turn help controls leukocyte trafficking and vasculature inflammation, potentially leading to cardiac repair by affecting the homing of hematopoietic progenitor cells [9,45].

## 3. miRNAs in Cardiovascular Diseases

**Figure 3:**
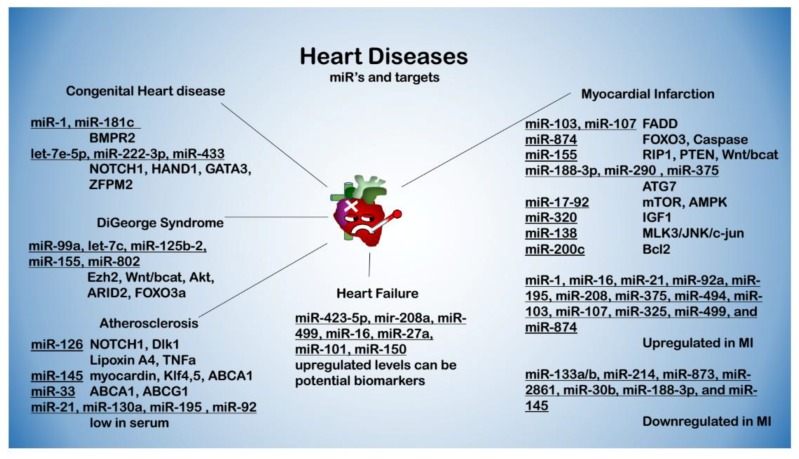
Major miRNAs (underlined) and targets in heart diseases (congenital heart diseases, DiGeorge syndrome, atherosclerosis, heart failure, myocardial infarction, and biomarkers).

Cardiovascular Diseases (CVDs) are the leading cause of morbidity and mortality in developed countries. Over the past two decades, much effort has been put into finding both the physiological fundamentals and molecular mechanisms of control for CVDs. Research on miRs in CVDs has pointed out the specificity of certain miRs and clusters to certain conditions. Here, we emphasize on some of the most important and clinically relevant miRs in conditions such as congenital heart disease (CHD), atherosclerosis, myocardial infarction (MI), severe coronary artery disease (CAD), and heart failure (HF) [4,9,15,33,57] (see Figure 3 and Supplementary Tables S1 and S2).

CHDs are the leading cause of prenatal deaths (~40%), while at the same time comprising the majority of all congenital malformations [58]. Reports from the Euro Heart Survey suggest that around 20% of patients with CHD undergo surgery or a catheter-based intervention, resulting in a major economic burden for the patient [15,33,59,60].

Ventricular septal defect (VSD) accounts for approximately 30–40% of CHDs, whereas atrial septal defects (ASD) are the cause of a smaller proportion. VSD and ASD can be viewed as a discontinuation in the septal wall dividing the ventricles and atria, respectively, of the heart. VSD can produce left ventricle overload, resulting in pulmonary hypertension [61,62]. VSD elevates levels of GJA1 and SOX9 overlap with reduced expression of miR-1-1, and elevates miR-181c [15]. MiR-1/181c additionally regulate the expression of Bone morphogenic receptor protein 2 (BMPR2), a key component in energy biogenesis [63,64]. Additional data form Li et al. showed that let-7e-5p, miR-222-3p, and miR-433 maybe the underlying cause for abnormalities since they target *NOTCH1, HAND1, GATA3*, and *ZFPM2*, resulting in altered morphogenesis and VSD [27].

DiGeorge syndrome is a direct result of deletion in region 8 of chromosome 22, leading to a loss-of-function mutation on *TBX1* and haploinsufficiency.

TBX1 is implicated in neural crest cell differentiation, in which mutations to the gene hinder correct formation of the outflow track [15]. In addition, this condition leads to DGCR8 downregulation and promotes an accumulation of both pri-*miRs* and pre-*miRs* [65].

Recent studies have shown 5 miRs to be directly correlated with Down syndrome (DS; CHD expectancy ~50–60%): miR-99a, let-7c, miR-125b-2, miR-155, and miR-802, all overexpressed in the heart [51]. MiR-99a has been associated with repression of cardiogenesis when expressed at an early stage, by regulating Smarca5. Let-7c was found to induce cardiogenesis, but only if expressed during mesoderm formation, thereby repressing the activity of *Ezh2* [66]. Additional studies in cancer have concluded that the loss of the let-7 family contributes to the upregulation of *Ezh2*, while miR-99a, typically found in patients with prostate cancer, can contribute to general cell proliferation [66]. Moreover, miR-155 overexpression can inhibit necrosis. Additional survival studies that suggest a mechanism via repression of receptor interacting protein 1 (RIP1) are independent of both the Wnt/β-catenin and Akt survival pathways [15,56,67]. MiR-155 is involved in many known aspects of regulatory biology: promoting cell proliferation by PTEN signaling pathway [29], promoting tumor growth by ARID2 repression [68], and regulating cell proliferation in glioma by targeting *FOXO3a* [69].

According to the AHA website (http://www.heart.org/, accessed on 20 April 2018) “Atherosclerosis is a big word for a big problem”. They define atherosclerosis as a buildup of fat deposits turning into plaques in the arteries. It is a multidimensional problem, not only dependent on the amount of circulating fat, but also on factors such as endothelial cell (EC) dysfunction, and vascular smooth muscle cell (VSMC) differentiation and inflammation. These buildups can lead to partial or full blockage and thus restrict blood flow, nutrition, and/or oxygen. Consequently, it is the initiator for many diseases, including CHD, angina, and carotid artery disease, amongst others [70]. A major component of atherosclerosis is EC dysfunction as a response to sheer stress. Schober et al. deduced that miR-126 directly affects vascular integrity, leading to the notion that miR-126-5p is mainly responsible for EC repair by inhibiting NOTCH1 and Dlk1, [71]. Additionally, a second isoform, miR-126-3p, is responsible for reducing inflammation signaling by promoting VCAM-1. By blocking these two mechanisms, atherosclerosis protection at the EC level is reduced [72,73]. Romano’s group uncovered the mechanics of understating the cascade activation of miR-126-5p by Lipoxin A4—a response via pro-inflammatory endothelial microvesicles packed with miR-126-5p, which antagonizes TNFα, leading to the upregulation of VCAM-1 and the downregulation of SPRED1 [71]. Another component of atherosclerosis is VSMC differentiation. MiR-145 deficiency has been shown to reduce the medial layer in arteries. In addition, the differentiation genes *myocardin, KLF4, KLF5*, calmodulin kinase, and cholesterol transporter *ABCA1* were found to be a direct target of miR-145 [74].

MiR-33a and miR-33b have been shown to regulate *ABCA1* and *ABCG1*, since they control the sterol regulatory element-binding proteins. Hence, their control can be a useful tool in potential therapies for dyslipidemia and atherosclerosis [75,76]. Hypertension or high blood pressure can lead to atherosclerosis; due to the added force at the artery walls, miR-145 and miR-143 seem to play an important role in high blood pressure, mainly targeting the angiotensin converting enzyme [4]. In addition, inhibition of miR-145 might improve diabetic resistance via nitric oxide [77]. We note that mouse studies have elucidated the role of miR-21, demonstrating reduced blood pressure in inverse correlation with miR-130a and miR-195, which have been shown to be positively upregulated in serum [78].

MI can be described as severe CAD or a myocardial cell death due to sustained ischemia. Patients with MI have shown higher levels of expression of miR-1, miR-133, miR-208, and miR-499 [79]. The dysregulation of all four miRs has been linked to MI [18,33,79,80]. MiR-208 by itself has been shown to be sufficient to induce heart hypertrophy as a response to overload while inducing β-MHC expression [81]. As mentioned in Sun et al., levels of expression of miR-1, miR-16, miR-21, miR-92a, miR-195, miR-208, miR-375, miR-494, miR-103, miR-107, miR-325, and miR-874 are appreciably upregulated in heart tissue MI, while the levels of tissue miR-133a/b, miR-214, miR-873, miR-2861, miR-30b, miR-188-3p, and miR-145 are decreased. These miRs contribute to the notion of specific spatial-time regulation, since many (if not all) are also involved in other conditions. In addition, protective signaling to reduce damage in the heart can be achieved by expression of miR-873 and miR-2861 [82]. The specificity required to precisely achieve protection in the midst of so much disruption can tell us more about the recurrent self-protective and pro-survival mechanisms present in the heart. It was mentioned that MI constitutes severe cell death, and cell death itself comes in 3 “flavors”: necrosis (necroptosis), autophagy, and apoptosis [82,83,84]. Each comes with a set of miR regulators acting on specific targets to both promote and inhibit each process.

Necrosis is a known form of death due to exacerbation in cellular or pathogenic damage. In cardiomyocytes, necrotic death induced by O_2_ elevates levels of miR-103 and miR-107, which act on the Fas-associated protein with death domain [85]. Meanwhile miR-874 expression can lead to necrosis by activating FOXO3a and Caspase-8 [86]. Recently, research has revealed a form of necrosis via programmed death: necroptosis. This process is initiated by TNF-α with directed interactions to RIP (1 and 3) proteins. Briefly, deficiency in interferon-β and MLK, as a result of TNFα/RIP, induce pyruvate dehydrogenase to induce what is now referred to as the necrosome (independent of caspase-8). However, this pathway can be mediated by activation of miR-874 [54,87]. Additionally, miR-155 can block RIP1 interaction, inhibiting the necrosome [29,56].

Autophagy is a highly conserved process of delivering intracellular components, including mitochondria and long-lived macromolecules, via a double-membrane structure, to the lysosomes for degradation [83]. When activated, miR-188-3p, miR-290, and miR-375 act as mediators reducing autophagitic activity by activating *ATG7*. ATG7 acts through a thiol–ester bond on the E1 activator to free ubiquitin molecules, beginning degradation [88]. At a molecular level, master regulators for autophagy are mTOR and AMPK, both regulated by miR-155 and the miR-17-92 complex. Above, it was mentioned that miR-155 could repress activation of the RIP complex by inhibition with PTEN, as well as interfering with the Wnt/β-catenin and the Akt-pro survival pathway [29,67]. Additionally, the miR-17-92 complex results in the downregulation of *mTOR* [31]. FOXO3a, a pro-autophagitic factor, is negatively regulated by both miR-212 and miR-132. Over expression of miR-212 and miR-132 significantly disturbs autophagy and results in drastic cardiac hypertrophy and heart failure [69,89]. Additionally, the energy sensing pathway of AMPK can be blocked by disruption of the α1 subunit (AMPKα1), and activation of miR-148b directly inhibits its expression [90], thereby blocking the entire AMPK assembly.

Apoptosis, or programed cell death, is a process driven by cell death receptors. Cascading signals are mediated by many pro- and anti-apoptotic signals—caspases, the Bcl-2 family, and p53 [83,91]. Hypoxia-inducible apoptosis is a condition in which a low oxygen concentration induces the overexpression of HIF-1; this initializes apoptotic conditions by inducing high concentrations of BNIP3 and causing stabilization of p53 [92,93,94]. MiR-138 exhibits a protective effect against hypoxia-induced apoptosis via the MLK3/JNK/c-jun pathway [95]. By downregulating JNK, p38, Bax, and *Caspase-3* levels, and upregulating *Bcl-2*, we can find an apoptotic for miR-320, namely IGF-1. Note that during inhibition of miR-320, the level of IGF1 mRNA is upregulated (Sun et al., 2017 [82]). Also, the anti-apoptotic signaling is the downregulation of miR-200c, as it increases levels of Bcl2 [96].

An important study by the American Heart Association and the American Stroke Association looked at cavernous malformations, which are best defined as circumscribed vascular lesions with thin-walled sinusoidal spaces lined with endothelial tissue and containing intravascular or intervascular calcifications [97]. Developmentally, these malformations control cardiac development via endothelial signaling of MEKK (MAPK/ERK pathway) and KLF [98]. PDCD10, a major role-playing factor in this condition, is heavily involved in cardiomyocyte autophagy. It has been shown to be susceptible to regulation by miR-613 by acting over *LXRα* [99], while *PDCD4* is a direct target of miR-155 [100].

Yan et al. describe HF as a terminal stage of most types of cardiovascular diseases that always leads to a negative prognosis. Their study on the clinical relevance of using circulating levels of miR-423-5p as a potential biomarker found that the standard marker is B type natriuretic peptide (BNP) [101]. Similarly, BNP in MI, miR-208b, and miR-499 do not represent optimal biomarkers, although they are under great scrutiny [82]. Meanwhile, research is being conducted to use miR-1 and miR-30a, since they play crucial roles in cardiac hypertrophy and apoptosis by targeting key molecules in the signaling pathways [102]. Nonetheless, the current consensus opinion is that there are no well-established biomarkers for HF.

## 4. Future Directions

The clinical potential underlying miRs can be seen as a great area of opportunity, both for their targeting and as biomarkers. miRs themselves are clearly expressed in a tightly regulated fashion throughout development and during organ maintenance. At the same time, miRs show some peculiarities in expression during pathological altered states. In the clinic, knowing and understanding these imbalances takes us a step forward.

Important elements for the usage or target of miRs should be summarized, as certain elements can be considered strengths or weaknesses. Consider the size of a mature miR, which varies around 22 bp and can be feasibly seen as a target of an antagonizing sequence (antagomiR or anti-miR), analogous to the mRNA outcompeting it and thereby not inhibiting transcription [24,67,75]. The remarkable feature here is that a single upstream target can determine the fate of a whole signaling pathway instead of targeting individual factors by knockdowns or having to obliterate by fully knocking out a gene, which is not always a viable solution. Counterintuitively, pairing a 22 bp fragment is relatively easy, but we need to consider the dynamics of targeting a miR to its mRNA. This relates to the “seed”, a 5 bp region at the 5′ end and up to 2 more adjacent basepairs, as using the seed alone is often not sufficient for pairing. Therefore, it is not uncommon for non-canonical pairing to occur [1]. Hence, the potential for silencing off targets exists, so sequences need to be fully vetted by bioinformatics systems [23]. Another aspect to consider is that miRs have a short half-life, hence modifications or high quantities are considered as a possibility when intended for therapeutics. Moreover, this might lead to toxicity issues and alterations of biological properties [48]. Alternatively, a different strategy all together is blocking miRs that counter an undesired effect. To this extent numerous miRs inhibitors have been designed with different adjustments within their structure, in order to enhance their function. Some of the most common include 2′-MOE (2′-*O*-methoxyethyl) and 2′-fluoro, as they exhibit higher affinity when compared to others [103].

Remarkably in blood and other body fluids, miRNAs are stable (28 h–5 days) [103], in part due to the fact they are protected from RNA-degrading molecules by their association to lipoproteins in extracellular vesicles [4]. An interesting limitation for detection of miRs in body fluids stems from the use of the well-defined oligonucleotides used for PCR; if the reactions are not under the precise conditions required for small nucleic acids (including the design of the oligonucleotides), it can easily lead to a large number of artifacts [104]. Nevertheless, in a clinical setting, circulating miR-screening, due to its sensibility, can be an advantageous tool to pair with traditional testing, thereby providing a better framework for diagnosis. MiRs as serum biomarkers have been rigorously reviewed [74,79,101,104,105]. Well-known markers for MI, such as those mentioned by Sun et al., serve as a perfect example of well-defined markers circulating in the plasma. They found miR-1, miR-16, miR-21, miR-92a, miR-195, miR-208, miR-375, miR-494, miR-103, miR-107, miR-325, miR-499, and miR-874 to be upregulated during an before an MI, while at the same time miR-133a/b, miR-214, miR-873, miR-2861, miR-30b, miR-188-3p, and miR-145 are downregulated [82]. Moreover, we have mentioned above that both atherosclerosis and HF also (although not well established) have biomarkers, such as miR-21, miR-130a, miR-195, miR-92 (atherosclerosis) and miR-423-5p, miR-208a, miR-499, miR-16, miR-27a, miR-101, and miR-150 (HF) [78] (see Table S2).

Attempts to direct mimetics and antagomiRs to a specific organ pose a fascinating challenge. A direct injection the antagomiR to the organ is usually the best route, and delivery systems range from the use of liposome vesicles, polymers, and other viral particles. Yet, for the patient, it can lead to high costs [106]. The first set of experiments using antagomiR reported a systematic reduction of miRs. Intravenous administrations were carried out against miR-16, miR-122, miR-192, and miR-194, resulting in a notable decline of the resultant miRNA levels in liver, lung, kidney, heart, intestine, fat, skin, bone marrow, muscle, ovaries, and adrenals [107]. Further studies have revealed the value of miRs to downregulate miRNAs in primates using locked nucleic acid (LNA) modification; these modifications connect the 2′-oxygen to the 4′-carbon of the ribose, resulting in a conformation which enhances mismatch discrimination [108]. The first set of around 20 clinical trials are currently underway using diverse delivery systems [17]. Nevertheless, the use of antagomiRs poses a number of potential setbacks, such as mistargets and delivery deficiencies. Yet, at the current speed of progress in science and technology, the opportunity to overcome them appears close. We expect the near future to hold many more key elements for personalized medicine.

Above, it was mentioned that miRs can have many potential targets. Consider, for example, the well-documented case of miR-29, which regulates fibrosis in the heart by targeting a whole set of components, such as elastins, fibrillins, and collagens—all components of the extra cellular matrix. Therefore, by using a well-designed antagomiR for miR-29, it has been possible to protect cardiomyocytes after damage from fibrotic remodeling [103]. Furthermore, during MI experiments it was found that antagomiRs for miR-92a and miR-320 reduced infarct size by contributing to recovery of blood vessels and reduction of apoptotic signals [57]. Meanwhile, Huang et al. considered the use of mesenchymal stem cells expressing miR-1. Elsewhere, it has been reported that miR-1 is sufficient to convert stem cell to cardiomyocytes. This experiment led to overall improvement of both cardiac function and a reduction in overall infarcted size [109]. Another set of experiments looked at miR-21. Dong et al. upregulated the expression of miR-21 by ischemic preconditioning—before MI—demonstrating considerable reduction in infarcted area. Moreover, the effect was even greater when injecting alongside miR-1 and miR-24 [57]. Experiments on atherosclerosis using antagomiRs (miR-33) showed a substantial elevation in high-density lipoprotein cholesterol (HDL) and reduction in very low-density lipoprotein cholesterol (VLDL). Additionally, LNA-AntagomiR (miR-122) showed promise in cholesterol reduction, while 2′-*O*-methoxyethyl phosphorothioate (miR-122) enhanced liver steatosis [104]. AntagomiR (miR-145) has been used in rodents to promote a reduction of plaque size and enhance vasculature. The results showed a marked reduction in plaque in aortic sinuses and the necrotic core, and increased collagen, which promoted contractile VSMC [74,104]. These studies demonstrated how, using an antagomiR or a miR-mimic, it is possible to achieve a desired outcome.

Since their initial discovery, small non-coding RNAs, have played an instrumental role in deciphering the nature and mechanics of human biology and its conditions. As we have seen in this overview of CVDs, they are instrumental both in the way they orchestrate through positive and negative feedback, and direct control. Ultimately, we see that they work in groups, many belonging to a family or super family of miRs. In the right balance, they are responsible for the correct cardiac environment. We can look forward to the next few years, in which technological advances will take us further in understanding and uncovering more insights into miRs, mimics, and antagomiRs. It is very likely that their localized use will be a common method of personalized treatment for many illnesses. In addition, we expect an even faster uptake in the number of clinics using screening methods to identify miR levels in patients as a way to easily access disease information.

## Figures and Tables

**Table 1:** Principal miRNA during Heart development and Embryonic Stem cell differentiation.

MicroRNA	Regulation	Targets	References
miR-1/-133	Proliferation and muscle growth	SRF, MEF2c, MyoD, Hand2, Myocardin	[23]
	Signal mesoderm formation	Twist	[34]
miR-1	Conduction	NCX1	[22]
	Signaling	Repression of HDAC4 Activation of MEF2	[34,35]
miR-1-2	Repolarization	Kcnd2	[26,27]
miR-15 family	Cell Cycle	Repression of HSP-20	[30]
miR-17-92 complex	Second heart field	BMP signaling, SMAD repression Isl1, Tbx1	[30]
	Signaling	Repression of PTEN	[9,29,30]
	Signaling	mTOR	[31]
miR-34a	Specification	NOTCH1, Dlk1, Jagged, Hey, Hes	[24,25]
miR-155-3p	Regulates	MEF2c, KRAS (activate contractile factors)	[20,34,35]
miR-208	Hypertrophy and muscle growth Myosin Heavy chain	a (208a), b (208b)	[32,33]
	Conduction	GATA4, CX40	
miR-199/-483	Signal mesoderm formation	Repression of Dlk-1	[34]
miR-200c	Cardiac TF Conduction system	GATA4, TBX5, SRF CACNA1C, KCNJ2, SCN5A	[36]
miR322/503	Mesoderm formation	Celf1	[37]
miR-1/-499	Electrical/conduction	Upregulates Kir2.1, Kv1.4, HERG, and DHPR Downregulates HCN4	[18,38]
miR-1/-133/-208/-499	Enhanced ES conversion		[33,37,39,40]

## Supplementary Materials

Supplementary materials can be found at http://www.mdpi.com/1422-0067/19/7/2075/s1.

## References

[1] Hydbring P., Badalian-Very G. (2014). Clinical applications of microRNAs. F1000Research.

[2] Wightman B., Ha I., Ruvkun G. (1993). Posttranscriptional regulation of the heterochronic gene lin-14 by lin-4 mediates temporal pattern formation in *C. elegans*. Cell.

[3] Zhang L., Hao C., Li J., Qu Y., Bao L., Li Y., Yue Z., Zhang M., Yu X., Chen H. (2017). Bioinformatics methods for identifying differentially expressed genes and signaling pathways in nano-silica stimulated macrophages. Tumour Biol..

[4] Romaine S.P.R., Tomaszewski M., Condorelli G., Samani N. (2015). MicroRNAs in cardiovascular disease: An introduction for clinicians. Heart.

[5] Li Y., Kowdley K.V. (2012). MicroRNAs in Common Human Diseases. Genom. Proteom. Bioinform..

[6] Sohel M.H. (2016). Extracellular/Circulating MicroRNAs: Release Mechanisms, Functions and Challenges. Achiev. Life Sci..

[7] Bartel D.P. (2009). MicroRNA Target Recognition and Regulatory Functions. Cell.

[8] Gulyaeva L.F., Kushlinskiy N.E. (2016). Regulatory mechanisms of microRNA expression. J. Transl. Med..

[9] Small E.M., Frost R.J.A., Olson E.N. (2010). MicroRNAs add a new dimension to cardiovascular disease. Circulation.

[10] Hunter M.P., Ismail N., Zhang X., Aguda B.D., Lee E.J., Yu L., Xiao T., Schafer J., Lee M.L.T., Schmittgen T.D. (2008). Detection of microRNA expression in human peripheral blood microvesicles. PLoS ONE.

[11] Lee Y., Jeon K., Lee J.-T., Kim S., Kim V.N. (2002). MicroRNA maturation: Stepwisee processing and subcellular localization. EMBO J..

[12] Garzon R., Marcucci G., Croce C.M. (2013). Targeting MicroRNAs in Cancer: Rationale, Strategies and Challenges. Nat. Rev. Drug Discov..

[13] Demongeot J., Glade N., Moreira A., Vial L. (2009). RNA relics and origin of life. Int. J. Mol. Sci..

[14] Chendrimada T.P., Gregory R.I., Kumaraswamy E., Cooch N., Nishikura K., Shiekhattar R. (2005). TRBP recruits the Dicer complex to Ago2 for microRNA processing and gene silencing. Nature.

[15] Smith T., Rajakaruma C., Caputo M., Emanueli C. (2015). MicroRNAs in congenital heart disease. Ann. Transl. Med..

[16] Alhendi A.M.N., Haider S., Jagannathan S., Anaissie E., Driscoll J.J. (2014). MicroRNA theragnostics for the clinical management of multiple myeloma. Leukemia.

[17] Chakraborty C., Sharma A.R., Sharma G., Doss C.G.P., Lee S.S. (2017). Therapeutic miRNA and siRNA: Moving from Bench to Clinic as Next Generation Medicine. Mol. Ther. Nucleic Acids.

[18] Fu J.-D., Rushing S.N., Lieu D.K., Chan C.W., Kong C., Wilson K.D., Chiamvimonvat N., Boheler K.R., Wu J.C., Hajjar R.J. (2010). Chiamvimonvat N, Li RA. Na^+^/Ca^2+^ exchanger is a determinant of excitation-contraction coupling in human embryonic stem cell-derived ventricular cardiomyocytes. Stem Cells Dev..

[19] Iyer D., Belaguli N., Flu M., Rowan B.G., Wei L., Weigel N.L., Booth F.W., Epstein H.F., Schwartz R.J., Balasubramanyam A. (2003). Novel Phosphorylation Target in the Serum Response Factor MADS Box Regulates. Biochemistry.

[20] Zheng G., Tao Y., Yu W., Schwartz R.J. (2013). Brief report: Srf-dependent MiR-210 silences the sonic hedgehog signaling during cardiopoesis. Stem Cells.

[21] Liu Y., Schwartz R.J. (2013). Transient Mesp1 expression: A driver of cardiac cell fate determination. Transcription.

[22] Tritsch E., Mallat Y., Lefebvre F., Diguet N., Escoubet B., Blanc J., De Windt L.J., Catalucci D., Vandecasteele G., Li Z. (2013). An SRF/miR-1 axis regulates NCX1 and Annexin A5 protein levels in the normal and failing heart. Cardiovasc. Res..

[23] Tian J., An X., Niu L. (2017). Role of microRNAs in cardiac development and disease. Exp. Ther. Med..

[24] Chaitra K.L., Ulaganathan K., James A., Ananthapur V., Nallari P. (2013). miRNA regulation during cardiac development and remodeling in cardiomyopathy. EXCLI J..

[25] Wu K.H., Xiao Q.R., Yang Y., Xu J.L., Zhang F., Liu C.M., Zhang Z.M., Lu Y.Q., Huang N.P. (2018). MicroRNA-34a modulates the Notch signaling pathway in mice with congenital heart disease and its role in heart development. J. Mol. Cell. Cardiol..

[26] Mishima Y., Stahlhut C., Giraldez A.J. (2007). miR-1-2 Gets to the Heart of the Matter. Cell.

[27] Li J., Dong X., Wang Z., Wu J. (2014). MicroRNA-1 in cardiac diseases and cancers. Korean J. Physiol. Pharmacol..

[28] Zhao Y., Ransom J.F., Li A., Vedantham V., von Drehle M., Muth A.N., Tsuchihashi T., McManus M.T., Schwartz R.J., Srivastava D. (2007). Dysregulation of Cardiogenesis, Cardiac Conduction, and Cell Cycle in Mice Lacking miRNA-1-2. Cell.

[29] Xu L., Len H., Shi X., Ji J., Fu J., Len H. (2017). MiR-155 promotes cell proliferation and inhibits apoptosis by PTEN signaling pathway in the psoriasis. Biomed. Pharmacother..

[30] Yan S., Jiao K. (2016). Functions of miRNAs during mammalian heart development. Int. J. Mol. Sci..

[31] Danielson L., Park D., Rotllan N., Chamorro-Jorganes A., Guijarro M.V., Fernandez-Hernando C., Fishman G.I., Phoon C., Hernando E. (2013). Cardiovascular dysregulation of miR-17-92 causes a lethal hypertrophic cardiomyopathy and arrhythmogenesis. FASEB J..

[32] Callis T.E., Pandya K., Hee Y.S., Tang R.H., Tatsuguchi M., Huang Z.P., Chen J.F., Deng Z., Gunn B., Shumate J. (2009). MicroRNA-208a is a regulator of cardiac hypertrophy and conduction in mice. J. Clin. Investig..

[33] Xin M., Olson E.N., Bassel-duby R. (2013). Mending broken hearts: Cardiac development as a basis for adult heart regeneration and repair. Nat. Rev. Mol. Cell Biol..

[34] Ivey K.N., Muth A., Arnold J., King F.W., Yeh R., Jason E., Hsiao E.C., Schwartz R.J., Conklin B.R., Harold S. (2009). MicroRNA Regulation of Cell Lineages in Mouse and Human Embryonic Stem Cells. Cell Stem Cell..

[35] Zhang S.X., Garcia-Gras E., Wycuff D.R., Marriot S.J., Kadeer N., Yu W., Olson E.N., Garry D.J., Parmacek M.S., Schwartz R.J. (2005). Identification of direct serum-response factor gene targets during Me_2_SO-induced P19 cardiac cell differentiation. J. Biol. Chem..

[36] Poon E., Hao B., Guan D., Li M., Lu J., Yang Y., Wu B., Wu S., Webb S., Liang Y. (2018). Integrated transcriptomic and regulatory network analyses identify microRNA-200c as a novel repressor of human pluripotent stem cell-derived cardiomyocyte differentiation and maturation. Cardiovasc. Res..

[37] Shen X., Soibam B., Benham A., Xu X., Chopra M., Peng X., Yu W., Bao W., Liang R., Azares A. (2016). miR-322/-503 cluster is expressed in the earliest cardiac progenitor cells and drives cardiomyocyte specification. Proc. Natl. Acad. Sci. USA.

[38] Lieu D.K., Fu J., Chiamvimonvat N., Tung K.W.C., McNerney G.P., Huser T., Keller G., Kong C.-W., Li R.A. (2013). Mechanism-Based Facilitated Maturation of Human Pluripotent Stem Cell-Derived Cardiomyocytes. Circ. Arrhythm. Electrophysiol..

[39] Christoforou N., Chellappan M., Adler A.F., Kirkton R.D., Wu T., Addis R.C., Bursac N., Leong K.W. (2013). Transcription Factors MYOCD, SRF, Mesp1 and SMARCD3 Enhance the Cardio-Inducing Effect of GATA4, TBX5, and MEF2C during Direct Cellular Reprogramming. PLoS ONE.

[40] Jayawardena T., Egemnazarov B., Finch E., Zhan L., Payne A., Pandya K., Zhang Z., Rosenberg P., Mirotsou M., Dzau V. (2013). MicroRNA-mediated in vitro and in vivo Direct Reprogramming of Cardiac Fibroblasts to Cardiomyocytes. Circ. Res..

[41] Guo X.-M. (2006). Creation of engineered cardiac tissue in vitro from mouse embryonic stem cells. Circulation.

[42] Srivastava D., Ieda M. (2013). Critical Factors for Cardiac Reprogramming. Circ. Res..

[43] Belian E., Noseda M., Abreu Paiva M.S., Leja T., Sampson R., Schneider M.D. (2015). Forward Programming of Cardiac Stem Cells by Homogeneous Transduction with MYOCD plus TBX5. PLoS ONE.

[44] Terentyev D., Belevych A.E., Terentyeva R., Martin M.M., Malana G.E., Kuhn D.E., Abdellatif M., Feldman D.S., Terry S., Gyorke S. (2015). Mir-1 overexpression enhances Ca^2+^ release and promotes cardiac arrhythmogenesis by targeting PP2A regulatory subunit B56α and causing camkii-dependent hyperphosphorylation of RyR2. Circ. Res..

[45] Poon K.S., Palanisamy K., Chang S.S., Sun K.T., Chen K.B., Li P.C., Lin T.C., Li C.Y. (2017). Plasma exosomal miR-223 expression regulates inflammatory responses during cardiac surgery with cardiopulmonary bypass. Sci. Rep..

[46] Bondue A., Blanpain C. (2010). MESP1. A Key Regulator of Cardiovascular Lineage Commitment. Circ. Res..

[47] Islas J.F., Liu Y., Weng K.-C., Robertson M.J., Zhang S., Prejusa A., Harger J., Tikhomirova D., Chopra M., Iyer D. (2012). Transcription factors ETS2 and MESP1 transdifferentiate human dermal fibroblasts into cardiac progenitors. Proc. Natl. Acad. Sci. USA.

[48] Joladarashi D., Thandavarayan R.A., Babu S.S. (2014). Small Engine, Big Power: MicroRNAs as Regulators of Cardiac Diseases and Regeneration. Int. J. Mol. Sci..

[49] Qiao G., Xia D., Cheng Z., Zhang G. (2018). Role of Sprouty1 (Spry1) in the pathogenesis of atrial fibrosis. Pathol. Res. Pract..

[50] Yang X., Gong Y., Tang Y., Li H., He Q., Gower L., Llaw L., Friesel R. (2013). Spry1 and Spry4 Differentially Regulate Human Aortic Smooth Muscle Cell Phenotype via Akt/FoxO/Myocardin Signaling. PLoS ONE.

[51] Zhao Y., Jaber V., Percy M.E., Lukiw W.J. (2017). A microRNA cluster (let-7c, miRNA-99a, miRNA-125b, miRNA-155 and miRNA-802) encoded at chr21q21.1-chr21q21.3 and the phenotypic diversity of Down’s syndrome (DS; trisomy 21). J. Nat. Sci.

[52] Li Y., Maegdefessel L. (2016). My heart will go on—Beneficial effects of anti-MiR-30 after myocardial infarction. Ann. Transl. Med..

[53] Tseliou E., de Couto G., Terrovitis J., Sun B., Weixin L., Marbán L., Marbán E. (2014). Angiogenesis, cardiomyocyte proliferation and anti-fibrotic effects underlie structural preservation post-infarction by intramyocardially-injected cardiospheres. PLoS ONE.

[54] Weber K., Roelandt R., Bruggeman I., Estornes Y., Vandenabeele P. (2018). Nuclear RIPK3 and MLKL contribute to cytosolic necrosome formation and necroptosis. Commun. Biol..

[55] Ling X., Yao D., Kang L., Zhou J., Zhou Y., Dong H. (2017). Involment of RAS/ERK1/2 signaling and MEF2C in miR-155-3p inhibition-triggered cardiomyocyte differentiation of embryonic stem cell. Oncotarget.

[56] Jablonska E., Gorniak P., Prusisz W., Kiliszek P., Szydlowski M., Sewastianik T., Bialopiotrowicz E., Polak A., Prochorec-Sobieszek M., Szumera-Cieckiewicz A. (2015). MiR-155 Amplifies AKT and NFkB Signaling By Targeting Multiple Regulators of BCR Signal in DLBCL. Blood.

[57] Schulte C., Zeller T. (2015). microRNA-based diagnostics and therapy in cardiovascular disease-Summing up the facts. Cardiovasc. Diagn. Ther..

[58] Bensemlali M., Bajolle F., Ladouceur M., Fermont L., Lévy M., Le Bidois J., Salomon L.J., Bonnet D. (2016). Associated genetic syndromes and extracardiac malformations strongly influence outcomes of fetuses with congenital heart diseases. Arch. Cardiovasc. Dis..

[59] Mozaffarian D., Benjamin E.J., Go A.S., Arnett D.K., Blaha M.J., Cushman M., Das S.R., De Ferranti S., Després J.P., Fullerton H.J. (2016). Heart disease and stroke statistics-2016 update a report from the American Heart Association. Circulation.

[60] Mercola M., Ruiz-lozano P., Schneider M.D. (2011). Cardiac muscle regeneration: Lessons from development Cardiac muscle regeneration: Lessons from development. Genes Dev..

[61] Bigdelian H., Sedighi M. (2017). The role of preoperative sildenafil therapy in controlling of postoperative pulmonary hypertension in children with ventricular septal defects. J. Cardiovasc. Thorac. Res..

[62] Lucchese G., Rossetti L., Faggian G., Luciani G.B. (2016). Long-Term Follow-Up Study of Temporary Tricuspid Valve Detachment as Approach to VSD Repair without Consequent Tricuspid Dysfunction. Tex. Heart Inst. J..

[63] Li J., Cao Y., Ma X., Wang H., Zhang J., Luo X., Chen W., Wu Y., Meng Y., Zhang J. (2013). Roles of miR-1-1 and miR-181c in ventricular septal defects.le. Int. J. Cardiol..

[64] Das S., Bedja D., Campbell N., Dunkerly B., Chenna V., Maitra A., Steenbergen C. (2014). miR-181c Regulates the Mitochondrial Genome, Bioenergetics, and Propensity for Heart Failure In Vivo. PLoS ONE.

[65] Landthaler M., Abdullah Y., Tuschi T. (2004). The human DiGeorge syndrome critical region gene 8 and its *D. melanogaster* homolog are required for miRNA biogenesis. Curr. Biol..

[66] Coppola A., Romito A., Borel C., Gehrig C., Gagnebin M., Falconnet E., Izzo A., Altucci L., Banfi S., Antonarakis S.E. (2014). Cardiomyogenesis is controlled by the miR-99a/let-7c cluster and epigenetic modifications. Stem Cell Res..

[67] Yan Q., Chen J., Li W., Bao C., Fu Q. (2016). Targeting miR-155 to Treat Experimental Scleroderma. Sci. Rep..

[68] Zhang L., Wang W., Li X., He S., Yao J., Wang X., Zhang D., Sun X. (2018). MicroRNA-155 promotes tumor growth of human hepatocellular carcinoma by targeting ARID2. Int. J. Oncol..

[69] Ling N., Gu J., Lei Z., Li M., Zhao J., Zhang H., Li X. (2013). microRNA-155 regulates cell proliferation and invasion by targeting FOXO3a in glioma. Oncol. Rep..

[70] Andrews J.P.M., Fayad Z.A., Dweck M.R. (2018). New methods to image unstable atherosclerotic plaques. Atherosclerosis.

[71] Codagnone M., Recchiuti A., Lanuti P., Pierdomenico A.M., Cianci E., Patruno S., Mari V.C., Simiele F., Di Tomo P., Pandolfi A. (2017). Lipoxin A4stimulates endothelial miR-126-5p expression and its transfer via microvesicles. FASEB J..

[72] Voora D. (2014). The Last Line of Defense Against Atherosclerosis. Sci. Transl. Med..

[73] Boon R.A., Dimmeler S. (2014). MicroRNA-126 in Atherosclerosis. Arterioscler. Thromb. Vasc. Biol..

[74] Faccini J., Ruidavets J.B., Cordelier P., Martins F., Maoret J.J., Bongard V., Ferrières J., Roncalli J., Elbaz M., Vindis C. (2017). Circulating MIR-155, MIR-145 and let-7c as diagnostic biomarkers of the coronary artery disease. Sci. Rep..

[75] Rayner K.J., Sheedy F.J., Esau C.C., Hussain F.N., Temel R.E., Parathath S., Van Gils J.M., Rayner A.J., Chang A.N., Suarez Y. (2011). Antagonism of miR-33 in mice promotes reverse cholesterol transport and regression of atherosclerosis. J. Clin. Investig..

[76] Rayner K.J., Esau C.C., Hussain F.N., Mcdaniel A.L., Marshall M., Van Gils J.M., Ray T.D., Sheedy F.J., Goedeke L., Liu X. (2012). Inhibition of miR-33a/b in non-human primates raises plasma HDL and reduces VLDL triglycerides Katey. Nature.

[77] Shiuchi T., Cui T.-X., Wu L., Nakagami H., Takeda-Matsubara Y., Iwai M., Horiuchi M. (2002). ACE Inhibitor Improves Insulin Resistance in Diabetic Mouse Via Bradykinin and NO. Hypertension.

[78] Yong W., Jin L. (2017). miRNA-145 is associated with spontaneous hypertension by targeting SLC7A1. Exp. Ther. Med..

[79] Wang G.-K., Zhu J.-Q., Zhang J.-T., Li Q., Li Y., He J., Qin Y.-W., Jing Q. (2010). Circulating microRNA: A novel potential biomarker for early diagnosis of acute myocardial infarction in humans. Eur. Heart J..

[80] Corsten M.F., Dennert R., Jochems S., Kuznetsova T., Devaux Y., Hofstra L., Wagner D.R., Staessen J.A., Heymans S., Schroen B. (2010). Circulating MicroRNA-208b and MicroRNA-499 reflect myocardial damage in cardiovascular disease. Circ. Cardiovasc. Genet..

[81] Kumarswamy R., Thum T. (2013). Non-coding RNAs in cardiac remodeling and heart failure. Circ. Res..

[82] Sun T., Dong Y.-H., Du W., Shi C.-Y., Wang K., Tariq M.-A., Wang J.-X., Li P.-F. (2017). The Role of MicroRNAs in Myocardial Infarction: From Molecular Mechanism to Clinical Application. Int. J. Mol. Sci..

[83] Chiong M., Wang Z.V., Pedrozo Z., Cao D.J., Troncoso R., Ibacache M., Criollo A., Nemchenko A., Hill J.A., Lavandero S. (2011). Cardiomyocyte death: Mechanisms and translational implications. Cell Death Dis..

[84] Graham R.M., Frazier D.P., Thompson J.W., Haliko S., Li H., Wasserlauf B.J., Spiga M.G., Bishopric N.H., Webster K.A. (2004). A unique pathway of cardiac myocyte death caused by hypoxia-acidosis. J. Exp. Biol..

[85] Wang J., Zhang X., Li Q., Wang K., Wang Y., Jiao J., Feng C., Zhou L., Gong Y., Zhou Z. (2015). MicroRNA-103/107 Regulate Programmed Necrosis and Myocardial Ischemia/Reperfusion Injury Through Targeting FADD. Circ. Res..

[86] Wang K., Liu F., Liu C., An T., Zhang J., Zhou L., Wang M., Dong Y., Li N., Gao J. (2016). The long noncoding RNA NRF regulates programmed necrosis and myocardial injury during ischemia and reperfusion by targeting miR-873. Cell Death Differ..

[87] Yang Z., Wang Y., Zhang Y., He X., Zhong C.-Q., Ni H., Chen X., Liang Y., Wu J., Zhao S. (2018). RIP3 targets pyruvate dehydrogenase complex to increase aerobic respiration in TNF-induced necroptosis. Nat. Cell Biol..

[88] Yamaguchi M., Satoo K., Suzuki H., Fujioka Y., Ohsumi O., Inagaki F., Noda N.N. (2018). Atg7 Activates an Autophagy-Essential Ubiquitin-like Protein Atg8 through Multi-Step Recognition. J. Mol. Biol..

[89] Sermersheim M.A., Park K.H., Gumpper K., Adesanya T.M.A., Song K., Tan T., Ren X., Yang J., Zhu H., Heart D. (2017). MicroRNA regulation of autophagy in cardiovascular disease. Front. Biosci. (Landmark Ed.).

[90] He X., Li C., Ke R., Luo L., Huang D. (2017). Down-regulation of adenosine monophosphate—Activated protein kinase activity: A driver of cancer. Tumor Biol..

[91] Samarel A.M. (2002). IGF-1 overexpression rescues the failing heart. Circ. Res..

[92] Greijer A.E., Van Der Wall E. (2004). The role of hypoxia inducible factor 1 (HIF-1) in hypoxia induced apoptosis. J. Clin. Pathol..

[93] Chu W., Wan L., Zhao D., Qu X., Cai F., Huo R., Wang N., Zhu J., Zhang C., Zheng F. (2012). Mild hypoxia-induced cardiomyocyte hypertrophy via up-regulation of HIF-1α-mediated TRPC signalling. J. Cell. Mol. Med..

[94] Ng K.M., Lee Y.K., Chan Y.C., Lai W.H., Fung M.L., Li R.A., Siu C.W., Tse H.F. (2010). Exogenous expression of HIF-1?? promotes cardiac differentiation of embryonic stem cells. J. Mol. Cell. Cardiol..

[95] Zhu H., Xue H., Jin Q.-H., Guo J., Chen Y.-D. (2017). MiR-138 protects cardiac cells against hypoxia through modulation of glucose metabolism by targetting pyruvate dehydrogenase kinase 1. Biosci. Rep..

[96] Chen Z., Zhang S., Guo C., Li J., Sang W. (2017). Downregulation of miR-200c protects cardiomyocytes from hypoxia-induced apoptosis by targeting GATA-4. Int. J. Mol. Med..

[97] Roach E.S., Golomb M.R., Adams R., Biller J., Daniels S., Ferriero D., Jones B.V., Kirkham F.J., Scott R.M., Smith E.R. (2008). Management of Stroke in Infants and Children a Scientific Statement from a Special Writing Group of the American Heart Association Stroke Council and the Council on Cardiovascular Disease in the Young. Stroke.

[98] Zhou Z., Rawnsley D.R., Goddard L.M., Pan W., Cao X.J., Jakus Z., Zheng H., Yang J., Arthur J.S.C., Whitehead K.J. (2015). The Cerebral Cavernous Malformation Pathway Controls Cardiac Development via Regulation of Endocardial MEKK3 Signaling and KLF Expression. Dev. Cell.

[99] Ou Z., Wada T., Gramignoli R., Li S., Strom S.C., Huang M., Xie W. (2011). MicroRNA hsa-miR-613 Targets the Human LXRα Gene and Mediates a Feedback Loop of LXRα Autoregulation. Mol. Endocrinol..

[100] Liu F., Song D., Wu Y., Liu X., Zhu J., Tang Y. (2017). MiR-155 inhibits proliferation and invasion by directly targeting PDCD4 in non-small cell lung cancer. Thorac. Cancer.

[101] Yan H., Zhang Y., Wang C., Qiu D., Zhou K., Hua Y., Li Y. (2017). miRNAs as biomarkers for diagnosis of heart failure. Medicine.

[102] Wong L.L., Wang J., Liew O.W., Richards A.M., Chen Y. (2016). MicroRNA and Heart Failure. Int. J. Mol. Sci..

[103] Kwekkeboom R.F.J., Lei Z., Doevendans P.A., Musters R.J.P., Sluijter J.P.G. (2014). Targeted delivery of miRNA therapeutics for cardiovascular diseases: Opportunities and challenges. Clin. Sci..

[104] Condorelli G., Latronico M.V.G., Cavarretta E. (2014). microRNAs in Cardiovascular Diseases Current Knowledge and the Road Ahead. J. Am. Coll. Cardiol..

[105] Gomes da Silva A.M., Silbiger V.N. (2014). miRNAs as biomarkers of atrial fibrillation. Biomarkers.

[106] Thomson D.W., Bracken C.P., Szubert J.M., Goodall G. (2013). On measuring miRNAs after transient transfection of mimics or antisense inhibitors. PLoS ONE.

[107] Krützfeldt J., Rajewsky N., Braich R., Rajeev K., Tuschl T., Manoharan M., Stoffel M. (2005). Silencing of microRNAs in vivo with “antagomirs”. Nature.

[108] Elmen J., Lindow M., Schutz S., Lawrence M., Petri A., Obad S., Lindholm M., Hedtjarn M., Hansen H., Berger U. (2008). LNA-mediated microRNA silencing in non-human primates. Nature.

[109] Huang F., Li M.L., Fang Z.F., Hu X.Q., Liu Q.M., Liu Z.J., Tang L., Zhao Y.S., Zhou S. (2013). Overexpression of MicroRNA-1 improves the efficacy of mesenchymal stem cell transplantation after myocardial infarction. Cardiology.

