# Supplementary material for: A MicroRNA Perspective on Cardiovascular Development and Diseases: An Update
Source: Int J Mol Sci. 2018 Jul 17;19(7):2075. doi: 10.3390/ijms19072075 (PMC6073753; doi:10.3390/ijms19072075)
Supplement: Supplementary file 1 [file ijms-19-02075-s001.zip › ijms-307897-SI.docx]

**Table S1.** Principal miRNA dysregulation during Cardiovascular Diseases**.**

| **MicroRNA** | **Disease** | **Regulation** | **Targets** | **References** |
| --- | --- | --- | --- | --- |
| miR-1/181c | Congenital Heart Disease | Proliferation | BMPR2, SOX9, GJA1 | [64,63] |
| let-7e-5p, miR-222-3p, miR-433 | Congenital Heart Disease | Morphogenesis | NOTCH1, HAND1, GATA3, ZFPM2 | [27] |
| miR-99a, let-7c, miR-125b-2, miR-155, miR-802 | DiGeorge Syndrome | Development, growth | Overexpressed in DS affected hearts | [51] |
| miR-99a | DiGeorge Syndrome | Cardiogenic repression | Smarc5 | [66] |
| let-7c | DiGeorge Syndrome | Cardiogenic induction | Ezh2 | [66] |
| miR-155 | DiGeorge Syndrome | Necrotic repression | RIP1 | [56,4,67] |
|  |  | Tumor growth signaling | ARID2 | [68] |
|  |  | Glioma targeting | FOXO3a | [69] |
|  |  | Proliferation | PTEN | [29,71] |
| miR-126 | Atherosclerosis | Proinflammatory response | Lipoxin A4, TNF-α, NOTCH1, Dlk1 | [71] |
| miR-155, let7c | Atherosclerosis/CAD | Proinflammatory response serves as Biomarker | AT1R, SOCS1 Oct4, Sox2 | [56,67,69,74] |
| miR-145 | Atherosclerosis | Targeting ACE | ACE enzyme | [4] |
|  |  | NO Regulation | Nitric Oxide |  |
|  |  | Artery volume reduction | myocardin, Klf4,5, ABCA1 | [74] |
| miR-33 | Atherosclerosis/dyslipidemia | Control | ABCA1, ABCG1 | [75,4] |
| miR-21, miR-130a, miR-195, miR-92 | Atherosclerosis | Biomarkers | low in Serum | [78] |
| miR-423-5p, mir-208a, miR-499, miR-16, miR-27a, miR-101, miR-150 | Heart Failure | Biomarkers | high in Serum | [78] |

**Table S2.** Principal miRNA dysregulation during Myocardial Infarction.

| **MicroRNA** | **Regulation** | **Targets** | **References** |
| --- | --- | --- | --- |
| miR-1, miR-133, miR-208, miR-499 | Heavily upregulated | Cardiac fibroblast reprograming network | [80,79,33] |
| miR-103, miR-107 | Necrosis | FADD | [86] |
| miR-874 | Necrosis | FOXO3a, Caspase | [86] |
| miR-155, miR-874 | Necrosis | RIP1, PTEN, Wnt/βcat | [54,87] |
| miR-188-3p, miR-290, miR-375 | Autophagy | ATG7 | [54,87] |
| miR-17-92 complex | Autophagy | mTOR | [54,87,31] |
| miR-212/132 | Autophagy | AMPK sensing | [69,89,90] |
| miR-320 | Apoptosis | IGF1 | [95] |
| miR-138 | Apoptosis | MLK3/JNK/c-Jun | [95] |
| miR-1, miR-16, miR-21, miR-92a, miR-195, miR-208, miR-375, miR-494, miR-103, miR-107, miR-325, miR-499, and miR-874 | Biomarker upregulated* | Upregulated in plasma | [82] |
| miR-133a/b, miR-214, miR-873, miR-2861, miR-30b, miR-188-3p, and miR-145 | Biomarker downregulated* | Downregulated in plasma | [82 |

* Most described miR´s as potential biomarkers, although full consensus established.
